# Immunotherapy and the Tumor Microenvironment in Brain Metastases from Non-Small Cell Lung Cancer: Challenges and Future Directions

**DOI:** 10.3390/curroncol32030171

**Published:** 2025-03-16

**Authors:** Meng Wang, Jihua Yang, Shuai Wang, Harjot Gill, Haiying Cheng

**Affiliations:** 1 Department of Oncology (Medical Oncology), Montefiore Medical Center, Albert Einstein College of Medicine, Bronx, NY 10461, USA; meng.wang@einsteinmed.edu (M.W.);; 2 Department of Pathology, Montefiore Medical Center, Bronx, NY 10461, USA

**Keywords:** brain metastases, non-small-cell lung cancer, immune system, tumor microenvironment, immune checkpoint inhibitors

## Abstract

Brain metastases (BMs) are a relatively common and severe complication in advanced non-small cell lung cancer (NSCLC), significantly affecting patient prognosis. Metastatic tumor cells can alter the brain tumor microenvironment (TME) to promote an immunosuppressive state, characterized by reduced infiltration of tumor-infiltrating lymphocytes (TILs), diminished expression of programmed death-ligand 1 (PD-L1), and changes in other proinflammatory factors and immune cell populations. Microglia, the resident macrophages of the brain, play a pivotal role in modulating the central nervous system (CNS) microenvironment through interactions with metastatic cancer cells, astrocytes, and infiltrating T cells. The M2 phenotype of microglia contributes to immunosuppression in BM via the activation of signaling pathways such as STAT3 and PI3K-AKT-mTOR. Recent advances have enhanced our understanding of the immune landscape of BMs in NSCLC, particularly regarding immune evasion within the CNS. Current immunotherapeutic strategies, including immune checkpoint inhibitors, have shown promise for NSCLC patients with BM, demonstrating intracranial activity and manageable safety profiles. Future research is warranted to further explore the molecular and immune mechanisms underlying BM, aiming to develop more effective treatments.

## 1. Introduction

Metastasis occurs when cancer cells spread from the primary tumor site and initiate new tumors in distant organs [1]. In the central nervous system (CNS), metastases primarily affect the brain parenchyma and leptomeninges, resulting in a poor prognosis characterized by high morbidity and mortality rates [2]. Globally, over 50% of brain metastases (BMs) originate from lung cancer [3]. While chemotherapy and radiotherapy have limited efficacy in treating BM, advancements in molecularly targeted therapies and immunotherapy show promising antitumor activity against BM and represent potential treatment strategies [4].

## 2. The Formation of Brain Metastases in NSCLC

**Figure 1:**
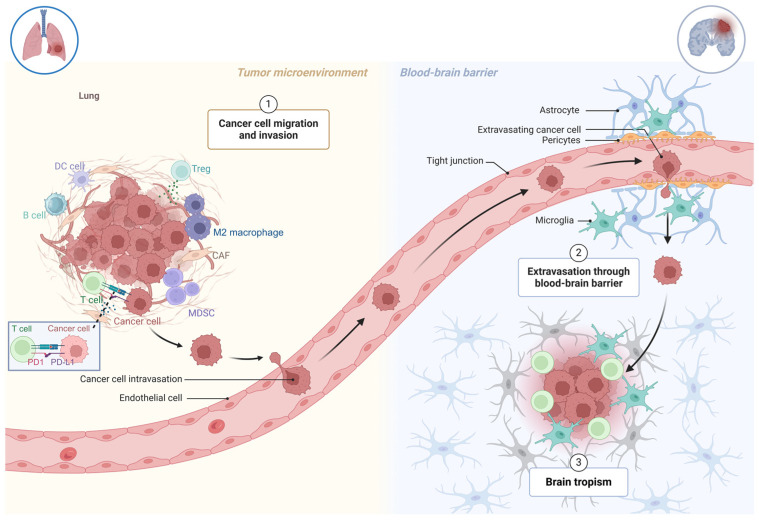
A summary of the non-small cell lung cancer brain metastasis. Created in BioRender. Wang, M. (2025) https://BioRender.com/e30a983 (accessed on 8 March 2025).

Cancer metastasis, a hallmark of malignant tumors, involves a complex, multistep process known as the invasion–metastasis cascade. During this progression, tumor cells detach from their primary site, enter the vasculature, survive in the bloodstream, invade distant organs, and adapt to new microenvironments, enabling their survival and proliferation in metastatic sites [5]. The nervous system, especially the brain, is a common metastatic destination for non-small cell lung cancer (NSCLC) [6]. Understanding brain metastases (BMs) necessitates a comprehensive perspective integrating immunity and inflammation. T cell infiltration is evident in BM, with higher densities of CD8+ and CD45RO+ T cells correlating with improved prognoses. Conversely, the presence of immunosuppressive CD4+CD25+FOXP3+ regulatory T cells is linked to poorer outcomes. Notably, compared to primary extracranial tumors, T cells in BM exhibit reduced infiltration, clonal expansion, and diversity [7]. The formation of BM in NSCLC underscores the critical role of immune responses and tumor cell interactions within the brain microenvironment. Tumor cells can penetrate and colonize the central nervous system, facilitated by significant alterations to the blood–brain barrier, which compromise its protective integrity and promote metastatic progression (Figure 1).

## 3. Blood–Brain Barrier

The blood–brain barrier (BBB) is a highly selective, semipermeable boundary that protects the CNS from harmful substances circulating in the bloodstream [8]. This unique anatomical structure comprises a continuous layer of endothelial cells connected by tight junctions (TJs), pericytes, and astrocytic end-feet, collectively forming the neurovascular unit (NVU). The BBB meticulously regulates the bidirectional movement of molecules between the vascular system and the brain, maintaining CNS homeostasis. During tumor metastasis, the BBB undergoes significant modifications that compromise its protective functions and facilitate tumor cell invasion. These alterations involve coordinated disruptions in the barrier’s integrity and function, including changes in tight junction proteins, basement membrane composition, and interactions between endothelial cells and astrocytes. Such modifications enhance BBB permeability, creating a microenvironment conducive to tumor cell penetration and colonization.

TJs proteins play a pivotal role in maintaining the structural and functional integrity of the BBB. These proteins form a molecular seal between adjacent endothelial cells, preventing paracellular diffusion and maintaining brain homeostasis. The primary transmembrane components of these complexes include claudins (particularly claudin-5), occludin, and junctional adhesion molecules (JAMs), supported by cytoplasmic adapter proteins such as zonula occludens-1 (ZO-1), which anchor TJs to the actin cytoskeleton. This intricate protein network establishes the BBB as a highly selective barrier, protecting the CNS from harmful substances and maintaining a stable environment for neural function [9]. Sun. ZW et al. have confirmed that a dysregulated Cldn5 level and repression of histone methylation, both as promoters, contributed to BBB dysfunction in male C57BL/6 mice [10]. The disruption of TJ proteins can significantly increase BBB permeability, altering the brain microenvironment. This disruption is a critical factor in various pathological conditions, including metastasis, where individual variations in BBB properties influence the ability of circulating tumor cells (CTCs) to penetrate and colonize brain tissue [8].

The basement membrane and astrocytic end-feet are equally vital to the NVU, contributing to BBB integrity and overall brain homeostasis [11]. The basement membrane, predominantly composed of type IV collagen, laminin, nidogen, and perlecan, provides structural support and regulates molecular trafficking through specific binding sites for endothelial cells and astrocytes [12]. This specialized extracellular matrix ensures mechanical stability and mediates cell–matrix interactions, essential for maintaining BBB integrity. Astrocytic end-feet completely enclose cerebral vasculature and facilitate nutrient exchange and waste removal, further supporting brain homeostasis. However, during metastatic progression, the interplay between the basement membrane, astrocytes, and endothelial cells undergoes significant disruption. Tumor cells orchestrate basement membrane degradation by upregulating matrix metalloproteinases (MMPs), particularly MMP-2 and MMP-9, in astrocytes. This enzymatic activity enhances BBB permeability, facilitating tumor cell invasion into the brain parenchyma.

## 4. Astrocytes

Astrocytes, the most abundant glial cells in the CNS, are involved in a wide range of functions, including synapse formation, metabolic support for neurons, regulation of BBB integrity, and modulation of behavior [13]. They maintain CNS homeostasis, ensuring effective neuronal communication and overall brain function. Recent advancements in single-cell RNA sequencing (scRNA-seq) and spatial transcriptomics have unveiled the heterogeneity of astrocyte populations, highlighting their active involvement in neuroinflammatory processes. These studies have demonstrated that astrocytes can adopt diverse activation states, contributing to the pathogenesis of various neurological diseases by modulating immune responses and maintaining CNS equilibrium [14,15]. In the context of brain metastasis, astrocytes are integral components of both primary and metastatic tumors, exhibiting dual roles by either inhibiting or promoting tumor progression [16].

Astrocytes regulate the tumor immune microenvironment through multiple pathways. Notably, the Janus Kinase (JAK)-Signal Transducer and Activator of Transcription (STAT) pathway is pivotal in astrocyte–tumor interactions. Astrocytes expressing phosphorylated STAT3 (pSTAT3) have been observed in brain metastases, where they impede CD8+ cytotoxic T cells from infiltrating cancer cells. This immunosuppressive effect is mediated through the upregulation of programmed cell death-1 ligand 1 (PD-L1), vascular endothelial growth factor-A (VEGF-A), lipocalin-2, and tissue inhibitor of metalloproteinases-1 (TIMP-1) [17,18]. Furthermore, STAT3+ reactive astrocytes in brain metastases exhibit increased expression of the CD74 ligand macrophage migration inhibitory factor (MIF), enhancing interactions with CD74+ microglia [19]. Cancer cells can reprogram astrocytes via the cyclic GMP-AMP synthase (cGAS)-Stimulator of Interferon Genes (STING) pathway by transferring 2′,3′-cyclic GMP-AMP (cGAMP), leading to the production of inflammatory cytokines such as interferon-α (IFN-α) and tumor necrosis factor-α (TNF-α) [20]. These cytokines activate STAT1 and the nuclear factor κB (NF-κB) signaling pathways in cancer cells, thereby supporting tumor metastasis. Understanding the multifaceted roles of astrocytes in tumor progression and the tumor immune microenvironment is crucial. Future research is warranted to unravel these intricate interactions and develop targeted therapies that modulate astrocyte activity, aiming to inhibit tumor growth and improve outcomes for patients with brain metastases.

## 5. Microglia

Microglia, the resident macrophages and principal immune cells of the CNS, fulfill a diverse array of functions beyond their conventional immune roles [21]. Together with astrocytes and other glial cells, such as oligodendrocytes, microglia contribute to establishing and maintaining a tightly regulated microenvironment crucial for neuronal function and overall brain homeostasis [22]. These cells play a vital role in continuously monitoring the brain and engaging in three primary functions. First, microglia detect changes in the microenvironment by expressing a variety of sensory genes. Second, they perform physiological housekeeping tasks, such as migrating to injury sites. This migration is regulated by localized cyclic adenosine monophosphate (cAMP), enabling microglia to sense environmental alterations and swiftly respond to molecular signals [23]. Third, microglia act as protectors against harmful stimuli, including pathogen-associated molecular patterns (PAMPs) and damage-associated molecular patterns (DAMPs). In this protective role, microglia release pro-inflammatory cytokines, such as tumor necrosis factor (TNF)-α, interleukin (IL)-1β, IL-16, and chemokines, to combat injurious conditions [24].

Microglia and macrophages are also pivotal in modulating CNS repair and regeneration. However, shifts in their phenotypes can be triggered by changes in the surrounding microenvironment [25]. Microglia typically adopt an activated state during disturbances. This activation can lead to increased levels of inflammatory cytokines and enhanced ability to stimulate a T cell-mediated antitumor response [26,27]. This pro-inflammatory activation, termed M1 polarization, is characterized by microglia’s defensive role in detecting and addressing environmental disturbances. In contrast, M2 polarization represents an anti-inflammatory phenotype that supports angiogenesis, tumor growth, and immunosuppression. M2-polarized microglia promote these effects by recruiting immune checkpoints, such as PD-L1 and V-domain Ig suppressor of T cell activation (VISTA) [28]. When co-culturing with NSCLC A549 cell lines to study biological behavior, M2 macrophages can stimulate A549 cell proliferation and tumor growth. In contrast, the M1 subtype inhibits the cells, triggering apoptosis and senescence through increased expression of DNA damage-induced proteins [29]. During chronic inflammation, microglia and astrocytes in the brain metastasis microenvironment may shift towards an anti-inflammatory phenotype, thereby facilitating tumor progression.

A significant driver of microglial polarization from the M1 to the M2 phenotype is the STAT3 signaling pathway. In the context of brain metastasis, microglia influence key processes, including BM cell migration across the endothelium and the modulation of gene expression. This pathway reduces ERK activity, a tumor growth inhibitor, while enhancing STAT3 phosphorylation, which promotes tumor proliferation [30]. Recent research highlighted the potential of targeting the IL-6R-JAK2-STAT3 signaling pathway in activated microglia as a novel strategy to inhibit brain metastasis in NSCLC [31].

In addition to STAT3, microglia/macrophages are involved in several other mechanisms during brain metastasis, notably the PI3K/Akt pathway. Evidence suggests that inhibiting this pathway in lipopolysaccharide (LPS)-activated microglia reduces the expression of pro-inflammatory factors [32]. In addition, the PI3K/Akt pathway study reported that it can be activated and contribute to the attenuation of brain damage. It can also upregulate the expression of immune checkpoints VISTA and PD-L1, inhibiting the T-cell immune response [33].

Together, microglia exhibit dual roles in brain metastasis by transitioning between pro-inflammatory (M1) and anti-inflammatory (M2) phenotypes. Critical signaling pathways, such as STAT3 and PI3K/Akt, play central roles in this polarization process, influencing both tumor growth and immune modulation. Understanding these mechanisms can provide valuable insights into the dynamic roles of microglia in brain metastasis and potential therapeutic targets.

## 6. NSCLC Tumor Microenvironment

NSCLC is characterized by a high tumor mutation burden (TMB), with metastatic lesions often harboring distinct oncogenic driver mutations compared to primary lung tumors [34]. These mutations can lead to the generation of neoantigens, which have the potential to elicit an antitumor immune response. The tumor microenvironment (TME) in NSCLC includes tumor-infiltrating lymphocytes (TILs) and tertiary lymphoid structures (TLSs), both of which are linked to a T helper 1 (Th1) and cytotoxic immune signature. These immune components have been associated with patient survival and therapeutic responses [35]. However, NSCLC also presents with an immunosuppressive TME maintained by regulatory T cells (Tregs). The high density of Tregs and a low CD8+ T cell-to-Treg ratio within the tumor tissue are strongly correlated with poor prognosis [36]. Recent research by Peng et al. highlighted that CD8+PD-L1+ TILs were associated with increased TMB but were embedded within an immunosuppressive TME. This underscores the complexity of immune interactions within NSCLC [37].

The immune response in NSCLC is often mediated by the PD-1/PD-L1 regulatory axis, where the interaction of PD-1 on effector T cells with PD-L1 expressed by tumor cells acts as an inhibitory signal [38]. This interaction promotes T cell exhaustion, limiting their antitumor activity. PD-L1 has emerged as a predictive biomarker for response to anti-PD-1/PD-L1 therapies in NSCLC [39]. Research by Liu ZC et al. has shown that circIGF2BP3 is CD8+ T cell-mediated and causes immune escape through decreased PD-L1 ubiquitination. It has the potential mechanism of PD-L1 regulation in NSCLC treatment [40]. Intriguingly, a recent study found that knocking down PD-L1 can reduce the expression of hexokinase-2, an enzyme critical for glycolysis, and inhibit the PI3K/AKT/mTOR and ERK pathways, which are key drivers of tumor growth and survival [41].

Immune checkpoint molecules such as CTLA-4 and PD-1, both members of the CD28 family, play central roles in tumor immunity [42]. Tumor-infiltrating Treg cells exhibit increased expression of surface molecules like CTLA-4, which are associated with T cell activation suppression. Studies suggest that anti-CTLA-4 monoclonal antibodies (mAbs) can enhance immune responses by increasing the activity of CD8+ and CD4+ T cells, further supporting their potential in immunotherapy for NSCLC [43].

Within the NSCLC TME, macrophages known as tumor-associated macrophages (TAMs) contribute significantly to immune evasion, cancer cell proliferation, invasion, and metastasis [44]. Similar to microglia in brain metastases, TAMs exhibit two main phenotypes with distinct functions. M1 macrophages are activated by pro-inflammatory stimuli, including Th1 cytokines such as interferon-gamma (IFN-γ), toll-like receptor (TLR) agonists like lipopolysaccharide (LPS), and the granulocyte–macrophage colony-stimulating factor (GM-CSF). These macrophages secrete pro-inflammatory cytokines such as tumor necrosis factor-alpha (TNF-α), IL-1, IL-12, and nitric oxide (NO), promoting an antitumor immune response [45]. In contrast, M2 macrophages, associated with type 2 helper T cells (Th2), secrete anti-inflammatory mediators such as IL-10 and transforming growth factor-beta (TGF-β) and release matrix metalloproteinases (MMPs) that contribute to tissue remodeling, angiogenesis, and tumor progression. The M2 phenotype facilitates tumor immune evasion, highlighting the dual role of TAMs in the NSCLC TME [46]. Overall, NSCLC is defined by a highly complex tumor microenvironment shaped by diverse cellular and immune interactions. While substantial progress has been made in understanding these dynamics, further research is essential to fully elucidate the mechanisms driving TME composition and to advance drug development targeting NSCLC.

## 7. Immunotherapy Targeting the BM

For advanced NSCLC without actionable genomic alterations (AGAs), first-line immunotherapy monotherapy in PD-L1 high NSCLC or combination with platinum-based chemotherapy, regardless of PD-L1 status, has become the standard of care (SOC). Here, we discuss the efficacy of immunotherapy, alone or in combination with chemotherapy, in NSCLC with BM (Table 1).

### 7.1. Immunotherapy Alone

A pooled analysis of KEYNOTE-001 (phase 1), 010 (phase 2/3), 024 (phase 3), and 042 (phase 3) studies evaluated 3170 patients with stage IV NSCLC and PD-L1 positive status, of whom 293 (9.2%) had BM. Patients received pembrolizumab monotherapy and were compared to chemotherapy except for KEYNOTE-001. These studies excluded patients with active BM or carcinomatous meningitis. Among patients with BM and PD-L1 ≥ 50%, pembrolizumab revealed a numerically longer median OS compared to chemotherapy (19.7 m vs. 9.7 m, HR 0.67, 95% CI 0.44–1.02). Similarly, in patients with BM and PD-L1 ≥ 1%, pembrolizumab showed a median OS of 13.4 months versus 10.3 months with chemotherapy (HR 0.83, 95% CI 0.62–1.10). However, the survival benefit in both PD-L1 groups was not statistically significant [47].

The IMPOWER-LUNG 1, a phase 3 randomized controlled trial, evaluated patients with advanced NSCLC without EFGR/ALK/ROS1 alterations and with PD-L1 ≥ 50%. Patients were randomized to receive first-line cemiplimab versus chemotherapy. The study enrolled 12.1% treated and clinically stable BM patients. Among this subgroup, the PFS and OS outcomes favored cemiplimab over chemotherapy [48].

In the CheckMate 227 trial, a phase 3 open-label, randomized controlled study, patients with stage IV or recurrent NSCLC without EGFR/ALK/ROS1 alterations were randomized in a 1:1:1 ratio to receive ipilimumab and nivolumab, nivolumab alone (for PD-L1 ≥ 1%), nivolumab plus chemotherapy (for PD-L1 < 1%), or platinum-based chemotherapy. A subgroup analysis of patients with and without BM at a 5-year follow-up demonstrated that the combination of ipilimumab and nivolumab significantly improved the median OS with 17.4 months compared to chemotherapy with 13.7 months (HR 0.63, 95% CI 0.42–0.92). Although the median intracranial PFS was not significantly different, the 5-year intracranial PFS rate was higher with dual immunotherapy (16%) compared to chemotherapy (6%). Notably, the PD-L1 ≥ 1% patients with BM showed a higher 5-year OS rate (27%, 95% CI 15–40) compared to chemotherapy (8%, 95% CI 3–18). And this led to FDA approval of the combination of ipilimumab and nivolumab as a first-line treatment for PD-L1 positive metastatic NSCLC [50].

Goldberg et al. provided additional insights into intracranial responses through a phase 2 trial that involved 42 advanced NSCLC patients with untreated or progressing BMs following radiation therapy and treated with pembrolizumab. Among patients with PD-L1-positive tumors, 29.7% (95% CI 15.9–47.0) achieved intracranial responses, while no IC responses were observed in the PD-L1-negative group [51].

A subgroup analysis of the phase 3 OAK trial assessed the efficacy of atezolizumab compared to docetaxel in advanced NSCLC patients with and without BM in the second-line setting. Among the 14% of patients with baseline BM, atezolizumab showed a numerically longer median OS compared to docetaxel (16.0 months vs. 11.9 months, HR 0.74, 95% CI 0.49–1.13) [52].

### 7.2. Immunotherapy Combined with Chemotherapy

A pooled subgroup analysis from the KEYNOTE-021 (nonsquamous), KEYNOTE-189 (nonsquamous), and KEYNOTE-407 (squamous) trials assessed the efficacy of pembrolizumab combined with chemotherapy versus chemotherapy alone in advanced chemotherapy-naive NSCLC patients with or without BM. Among the 1298 patients enrolled, 171 (13.2%) had baseline BM. In patients with BM, pembrolizumab plus chemotherapy demonstrated improved systemic ORR (39.0%) versus chemotherapy (19.7%). Significantly longer median OS was also observed in the pembrolizumab plus chemotherapy arm compared to chemotherapy alone (18.8 months vs. 7.6 months, HR 0.48, 95% CI 0.32–0.70). PFS was also significantly improved in the combination therapy with 6.9 months compared to chemotherapy with 4.1 months (HR 0.44; 95% CI 0.31–0.62). Notably, the PFS benefits of pembrolizumab plus chemotherapy were consistent across all PD-L1 expression subgroups [54].

CheckMate 9LA is a phase 3 randomized controlled trial evaluating the efficacy of the combination of ipilimumab plus nivolumab plus chemotherapy compared to chemotherapy alone as first-line treatment in metastatic or recurrent NSCLC without EGFR/ALK alterations. The study demonstrated significant OS benefits that favor the combination therapy which led to FDA approval in 2020. A subgroup analysis revealed that among the 14% of patients with baseline BM, the combination therapy significantly improved median OS compared to chemotherapy alone (19.3 months vs. 6.8 months, HR 0.45, 95% CI 0.29–0.70). PFS also favored the combination therapy arm over the chemotherapy arm. Intracranial activity was assessed, showing a higher intracranial ORR in the combination therapy arm (39.2% vs. 20.0%). Additionally, intracranial median PFS (11.4 months vs. 4.6 months, HR 0.42, 95% CI 0.26–0.68) was significantly longer in the combination therapy arm compared to chemotherapy alone. Furthermore, for patients with baseline BMs, fewer patients in the combination group developed new BMs compared to the chemotherapy group (20% vs. 30%, respectively). And similarly, fewer new BMs developed in the combination arm in those without baseline BM (3.2% vs. 3.6%) [55].

The phase II Atezo-Brain trial, a single-arm study, evaluated the combination of atezolizumab, carboplatin, and pemetrexed in patients with advanced non-squamous NSCLC without EGFR/ALK alterations and with untreated, asymptomatic BMs. Among the 40 enrolled patients, the systemic and intracranial response rates were 45% and 42.7%, respectively, with no significant differences in ORR observed across different PD-L1 expression groups. The median PFS was 8.9 months (95% CI 6.7–13.8), while the median OS was 11.8 months (95% CI 7.6–16.9). The intracranial PFS was 6.9 months (95% CI 4.7–11.9) [56].

Notably, no new safety concerns were identified in NSCLC patients with BM treated with ICI alone or in combination with chemotherapy. Several studies, including KEYNOTE 189, KEYNOTE 407, Goldberg et al., and Atezo-Brain, have investigated ICIs in NSCLC patients with untreated BMs. These trials demonstrated intracranial responses, suggesting that upfront systemic therapy incorporating ICIs could be a viable treatment option for this vulnerable population [51,54,56].

### 7.3. Limitation of ICI and Future Strategies

Although ICI has demonstrated some efficacy in managing NSCLC with BMs in several pivotal studies, this population remains significantly underrepresented. This is largely due to the exclusion of active or untreated BMs in many trials and the lack of prioritization of BMs as evaluable lesions in trial design. Notably, several studies evaluating patients with untreated BM [51,56], showed comparable IC efficacy to those with treated BM, demonstrating a promising frontline strategy in this challenging population. Moreover, in trials that included and evaluated patients with BMs, the efficacy of IC ORR remained below 50%, underscoring an ongoing unmet need. The combination of ICI with brain radiotherapy or other targeted therapies has evolved as a new strategy to overcome the low IC efficacy of ICI or ICI resistance. In a meta-analysis, ICI+ brain radiotherapy demonstrated superior efficacy [57]. Although the sequence is to be determined, some studies suggested the concurrent model might be the optimal approach [57,58]. ICI plus the anti-VEGF antibody has evolved as a new frontline method. It was previously evaluated in some trials including IMPOWER 150 [59] and the TASUKI-52 trial [60], demonstrating superior PFS (both) and OS (IMPOWER 150) when adding ICI (atezolizumab in IMPOWER 150 and nivolumab in TASUKI-52, respectively) to bevacizumab, carboplatin, and paclitaxel. More recently, the phase III HARMONI-2 trial showed that first-line ivonescimab, a bispecific antibody targeting PD-1 and VEGF, significantly improved PFS over pembrolizumab (11.14 m vs. 5.82 m; HR 0.51; 95% CI0.38–0.69; *p* < 0.0001) in advanced NSCLC with a positive PD-L1 score. This benefit was consistent in patients with and without BMs [61]. The IC activity has been evaluated in the phase II trials AK112-201 and AK112-202, and showed a combined IC ORR of 34% [62]. Novel target agents are undergoing exploration in clinical trials including LAG-3, TIM-3, TIGIT [63], and STING (NCT02675439, NCT03172936).

## 8. Targeted Therapies in NSCLC with BM

Targeted therapies have become the SOC for advanced NSCLC with AGAs, particularly for patients with BMs, which are commonly observed across molecular subtypes, with incidence rates ranging from 20% to 50% [64]. Given the high BM involvement, the development of TKIs with strong IC activity has been critical in improving outcomes.

### 8.1. EGFR

In *EGFR*-mutated NSCLC, osimertinib, a third-generation *EGFR* TKI, has remained the standard first-line treatment since 2018, and demonstrated a robust IC ORR of 91% in the FLAURA trial [65]. The FLAURA 2 trial built on these findings by adding platinum-based chemotherapy to osimertinib, showing a significant improvement in (PFS for patients with baseline BM (24.9 vs. 13.8 m) [66]. Meanwhile, the MARIPOSA trial evaluated a novel combination of amivantamab (an *EGFR-MET* bispecific antibody) and lazertinib (third-generation *EGFR* TKI), which demonstrated superior PFS over osimertinib alone in patients with BM (18.3 vs. 13.0 m) [67]. Currently, all of the three regimens have been approved by the FDA for first-line management in *EGFR*-mutated advanced NSCLC.

### 8.2. ALK

For *ALK*-positive NSCLC, second- and third-generation *ALK* TKIs have dramatically improved IC control compared to the first-generation TKI crizotinib, which has limited CNS penetration. Alectinib, brigatinib, and ensartinib are currently FDA-approved second-generation *ALK* TKIs with IC ORR 63–78% [68,69,70]. The third-generation *ALK* TKI lorlatinib has shown exceptional CNS efficacy. Lorlatinib achieved an IC ORR of 60%, with a complete response (CR) rate of 49% and a median PFS that was not reached in patients with BM. Remarkably, the 5-year IC PFS rate with lorlatinib was 92%, making it the leading option for *ALK*-rearranged NSCLC with BM [71].

### 8.3. ROS1

Crizotinib was the first approved *ROS1* TKI but has limited CNS activity [72]. Entrectinib has emerged as a more effective option for CNS disease, demonstrating a 52.2% IC ORR and median IC PFS of 8.3 months from pooled phase I/II studies [73]. More recently, repotrectinib, a next-generation *ROS1* TKI, has shown promising results, achieving an 89% IC ORR in *ROS1* TKI-naïve patients and 38% in patients previously treated with an *ROS1* TKI, according to the TRIDENT-1 trial [74].

### 8.4. Other Targeted Therapies

Beyond *EGFR*, *ALK*, and *ROS1*, several other targeted therapies have shown IC activity in NSCLC. RET fusion-positive patients benefit from selpercatinib [75] and pralsetinib [76], with IC ORRs of 82% and 70%, respectively. For KRAS G12C mutations, sotorasib and adagrasib have modest IC efficacy, with adagrasib achieving around 33–42% IC ORR [77,78,79]. *MET* exon 14 skipping patients respond to capmatinib [80] and tepotinib [81], with IC ORRs of 57% and 66.7%. For NTRK fusions, larotrectinib [82] and entrectinib [83] show promising IC responses. These newer therapies expand options for NSCLC patients with BM, but further studies are needed to optimize BM-specific management.

## 9. Conclusions and Perspectives

NSCLC remains one of the most prevalent and challenging cancers globally. NSCLC is also the leading cause of brain metastases (BMs), which present a distinct and complex tumor microenvironment. When BMs occur, unique features such as the blood–brain barrier, specialized extracellular matrix (ECM) composition, and tissue-resident cells interact to facilitate blood-derived immune cell infiltration and ECM remodeling, establishing a TME distinct from that of primary NSCLC. This specialized microenvironment poses significant challenges for treatment but also offers opportunities for targeted therapeutic interventions. The integration of conventional therapies with immunotherapy has already demonstrated promise, highlighting the critical role of the TME in modulating responses to immunotherapy. Future research into the molecular and cellular mechanisms underlying BM formation and progression is essential for advancing more effective treatment strategies and shaping a more personalized and impactful therapeutic landscape.

## Figures and Tables

**Table 1:** Clinical trials evaluating ICI with or without chemotherapy in advanced NSCLC with BM.

Trial	Type	Treatment	BM Eligibility	Patient Eligibility	Patient Number/BM Number	Systemic Outcome in Patients with BM	IC Outcome
Pooled analysis of KEYNOTE-001, -010, -024, -042 [47]	KEYNOTE-001 phase 1; KEYNOTE-010 phase 2/3; KEYNOTE -024 and -042 phase 3	Pembro vs. Chemo	No active BM, no carcinomatous meningitis	Previously treated and treatment naïve, PD-L1 positive. No *EGFR*/*ALK* alteration, or failed *EGFR* or *ALK* TKI (KEYNOTE 001 and KEYNOTE 010)	3170/293	PD-L1 ≥ 50% and BM: ORR: 33.9% vs. 14.6% mPFS 4.1 m vs. 4.6 m (HR 0.70, 95% CI 0.47–1.03) mOS 19.7 m vs. 9.7 m (HR 0.67, 95% CI 0.44–1.02) PD-L1 ≥ 1% and BM: ORR: 26.1% vs. 18.1% mPFS 2.3 m vs. 5.2 m (HR 0.96, 95% CI 0.73–1.25) mOS 13.4 m vs. 10.3 m (HR 0.83, 95% CI 0.62–1.10)	/
IMPOWER-Lung 1 [48,49]	Phase 3, randomized, controlled study	CEMI vs. Chemo	Treated and clinically stable BM	Advanced NSCLC with PD-L1 ≥ 50%, no *EGFR*/*ALK*/*ROS1* alterations	710 enrolled, 563 PD-L1 ≥ 50%/68	mPFS 10.4 m vs. 5.3 m (HR 0.45, 95% CI 022–0.92) mOS 18.7 m vs. 11.7 m (HR 0.17, 95% CI 0.04–0.76)	/
CheckMate-227 part 1 [50]	Phase 3, open label, randomized controlled study	Ipi + Nivo vs. Chemo	Treated and asymptomatic BM	Stage IV or recurrent NSCLC, treatment-naïve, no *EGFR*/*ALK* alterations	1739/202	ORR 32% vs. 26% mPFS 5.4 m vs. 5.8 m (HR 0.77, 95% CI 0.51–1.15) mOS 17.4 m vs. 13.7 m (HR 0.63, 95% CI 0.42–0.92)	IC PFS 8.6 m vs. 8.7 m (HR 0.82, 95% CI 0.52–1.30) 5-year IC PFS 16% vs. 6% New BM: 4% vs. 20%
Goldberg et al. [51]	Phase 2, open label, single arm	Pembro	Untreated or progressing after RT; no neurologic symptoms or steroid requirement	Stage IV NSCLC	42/42 (cohort 1 PD-L1 ≥ 1%: 37; cohort 2 PD-L1 < 1% or unevaluable: 5)	Cohort 1: mPFS 2.3 m (95% CI 1.9-NE) mOS 9.9 m (95% CI 7.5–29.8)	Cohort 1: IC ORR 29.7% (95% CI 15.9–47.0) Cohort 2: IC ORR 0
OAK study [52,53]	Phase 3, open label, randomized controlled study	Atezo vs. docetaxel	Treated, asymptomatic, supratentorial BM	Advanced NSCLC previously treated with platinum-based Chemo	850/123	mOS 20.1 m vs. 11.9 m (HR 0.54, 95% CI 0.31–0.94)	IC PFS NR vs. 9.5 m (HR 0.38, 95% CI 0.16–0.91, *p* = 0.024) IC OS 16.0 m vs. 11.9 m (HR 0.74, 95% CI 0.49–1.13, *p* = 0.16)
Pooled analysis of KEYNOTE-021, -189, -407 [54]	KEYNOTE-021 phase 2; KEYNOTE-189 and -407 phase 3	Pembro + Chemo vs. Chemo	Treated or untreated (KEYNOTE-189 and KEYNOTE-407 only) stable BM	Stage IIIB or IV (KEYNOTE-021 cohort G), Stage IV (KEYNOTE-189 and -407) nonsquamous without *EGFR*/*ALK* alteration (KEYNOTE-021 cohort G and KEYNOTE-189) or squamous (KEYNOTE-407), chemotherapy naïve NSCLC	1299/171	ORR 39.0% vs. 19.7% mPFS 6.9 m vs. 4.1 m (HR 0.44, 95% CI 0.31–0.62) mOS 18.8 m vs. 7.6 m (HR 0.48, 95% CI 0.32–0.70)	/
CheckMate 9LA [55]	Phase 3, open label, randomized controlled study	Ipi + Nivo + Chemo vs. Chemo	Treated and asymptomatic BM	Stage IV or recurrent NSCLC without *EGFR*/*ALK* alterations	719/101	ORR 43% vs. 24% mPFS 9.7 m vs. 4.1 m (HR 0.44, 95% CI 0.28–0.69) mOS 19.3 m vs. 6.8 m (HR 0.45, 95% CI 0.29–0.70)	IC ORR 39% vs. 20% IC PFS 11.4 m vs. 4.6 m (HR 0.42, 95% CI 0.26–0.68) New BM in pt with baseline BM: 20% vs. 30% New BM in pt without baseline BM: 3.2% vs. 3.6%
Atezo-Brain [56]	Phase 2, single arm	Atezo + c arboplatin + pemetrexed	Untreated and asymptomatic BM	Advanced nonsquamous NSCLC with BM, no *EGFR*/*ALK* alterations	40/40	ORR 45% (95% credibility interval Crl 28.1–57.9) mPFS 8.9 m (95% CI 6.7–13.8) mOS 11.8 m (95% CI 7.6–16.9)	IC ORR 42.7% (95% Crl 28.1–57.9) IC PFS 6.9 m (95% CI 4.7–11.9)

NSCLC: non-small cell lung cancer; BMs: brain metastases; Pembro: pembrolizumab; CEMI: Cemiplimab; Atezo: atezolizumab; Ipi: ipilimumab; Nivo: nivolumab; Chemo: chemotherapy; IC: intracranial; mPFS: median progression-free survival; mOS: median overall survival; ORR: objective response rate; NR: not reached; RT: radiotherapy.

## Data Availability

The supporting data are not publicly available due to research participant privacy restrictions.
